# Incision-triggered neuroimmune remodeling of nociceptor ion channels: from postoperative inflammation to pain chronification

**DOI:** 10.3389/fimmu.2026.1953337

**Published:** 2026-10-06

**Authors:** Xinye Song, Miao Feng, Jiachen Hao, Yong Luan

**Affiliations:** Department of Anesthesiology, The First Affiliated Hospital of Dalian Medical University, Dalian, Liaoning, China

**Keywords:** chronic postsurgical pain, macrophages, neuroimmune interactions, nociceptors, postoperative inflammation, sex-related biology, suzetrigine, voltage-gated sodium channels

## Abstract

Chronic postsurgical pain (CPSP) arises after a known tissue insult, yet no intervention reliably prevents its development. This Review examines whether incision-triggered inflammation remodels nociceptor voltage-gated ion channels in ways that sustain peripheral input and favor pain chronification. We integrate human genetic and clinical evidence, incision-specific perturbation studies, functional work in non-surgical models, and expression-level associations distinguishing mechanistic strength from directness to CPSP. We focus on NaV1.7, NaV1.8, NaV1.9, and CaV3.2 within a broader network that includes peripheral transducing channels whose gating, trafficking, and abundance can be altered by TNF-α, IL-1β, IL-6, chemokines, p38 MAPK, NF-κB, and gp130/JAK signaling. Direct incision evidence now includes antisense and pharmacologic studies linking TNF-α/p38/NF-κB signaling to NaV1.8 and NaV1.9 expression and acute hypersensitivity, as well as single-cell maps showing insult-specific macrophage and neutrophil programs and protective immune-derived signals. Evidence from rare channelopathies does not establish population-level CPSP susceptibility. However, no human study has demonstrated the complete sequence from a defined postoperative immune signal to altered channel function and persistent pain. Sex, age, and hormonal context are plausible modifiers, but the strongest mechanistic evidence remains model-dependent and preclinical. Selective NaV1.8 inhibition validates peripheral channel tractability for acute pain but not prevention of CPSP. We therefore propose a testable, evidence-calibrated framework in which pathogenic and resolving immune programs act across peripheral, ganglion, and spinal compartments, and we define the experiments, biomarkers, and target–time–population alignment needed to determine whether this framework can guide mechanism-based prevention.

## 1 Introduction

CPSP affects a substantial minority of patients who undergo common operations. It can persist after tissue healing, contributing to disability, prolonged analgesic use, and healthcare burden (1). Unlike most chronic pain conditions, surgery has a known initiating event. This feature allows the transition to be observed prospectively and potentially modified (2).

Pain chronification is an active process, not simply the persistence of acute nociception. A transient inflammatory insult can induce hyperalgesic priming and amplify later responses in selected primary afferents (3, 4). Peripheral and central sensitization may then overlap with neuroinflammation, failed resolution, and epigenetic plasticity (5, 6). These processes, however, are often studied in separate experimental literatures. This separation obscures the causal steps most relevant after surgery.

This review examines neuroimmune regulation of nociceptor voltage-gated ion channels as a candidate link between postoperative inflammation and persistent excitability. Rare human channelopathies establish that sodium-channel dysfunction can alter nociception, especially through NaV1.7 (7–9), but do not establish the contribution of these variants to CPSP in the general population. Experimental studies show that IL-1β, TNF-α, and p38 MAPK can alter sodium-channel inactivation, phosphorylation, current density, and membrane trafficking (10–14). However, most of these mechanisms were established outside surgical models.

Broad reviews have recently surveyed neuroimmune mechanisms and immunotherapeutic prospects across chronic pain syndromes, and a separate review has focused on epidermal growth factor receptor signaling in postoperative pain (15, 16). The present Review addresses a narrower unresolved question: whether immune signals generated by incision functionally remodel a defined nociceptor ion-channel network and thereby contribute to chronification. Its novelty is the combination of incision specificity, channel-level functional endpoints, explicit evidence grading, and consideration of sex-, age-, and hormone-related modifiers.

Two developments further increase the clinical relevance of this question. First, preclinical studies indicate that immune and channel-proximal mechanisms can vary with sex (17, 18). Second, selective NaV1.8 inhibition has produced acute analgesia in surgical trials (19, 20). We therefore ask what is established after incision and what is extrapolated from other pain states. We also examine how sex-related biology may modify the framework and which experiments could support prevention trials.

We use the term neuroimmune–channel framework to denote a set of testable relationships, not a pathway already established in patients. **Figure 1** presents the framework, while **Table 1** grades its major links by model, directness to surgery, functional endpoint, and causal strength.

**Figure 1:**
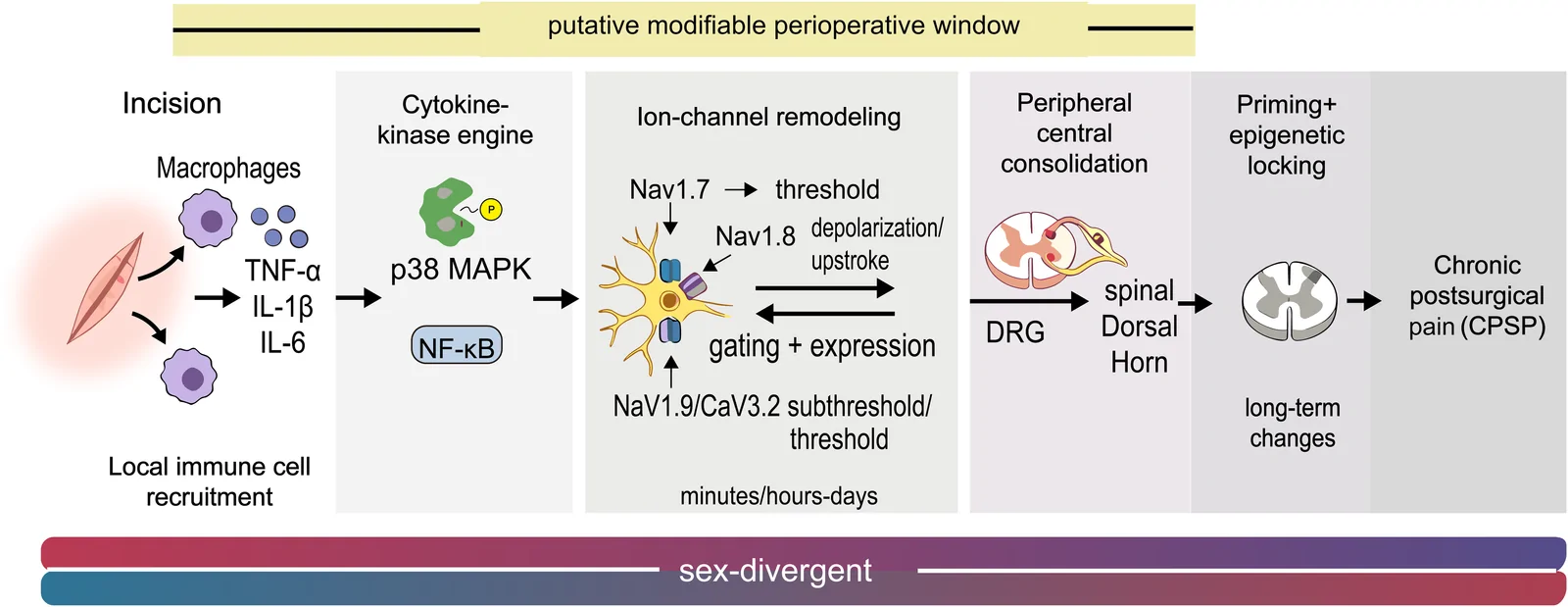
Evidence-calibrated framework for neuroimmune regulation of nociceptor ion channels after incision. The shaded interval denotes a putative modifiable period spanning the perioperative and early subacute phases, not a proven window of reversibility. The sex-related band represents model-dependent biological modifiers, not a binary human pathway.

**Table 1:** Evidence map for neuroimmune regulation of nociceptor ion channels.

Mechanistic claim	Model/species	Directness to surgery	Functional endpoint	Evidence class	Main limitation	Ref.
Incision evokes guarding and hypersensitivity	Plantar incision; rat	Direct surgical model	Behavioral phenotype	Model validation	Acute model; no channel endpoint	(56)
Incision evokes insult-specific macrophage and neutrophil programs; immune-derived thrombospondin-1 limits nociceptor sensitization	Skin incision and comparator injuries; mouse	Direct surgical model	Single-cell immune signatures, ligand–receptor interactome, and nociceptor sensitization	Cell-resolved discovery with functional validation	Acute cutaneous model; no direct NaV electrophysiology or chronic endpoint	(26)
TNF-α/p38 MAPK/NF-κB signaling increases DRG NaV1.8 and NaV1.9 transcripts after incision	Plantar incision; male mouse	Direct surgical model	Channel transcripts and mechanical hypernociception	Antisense knockdown plus pathway pharmacology	Mostly preventive treatment; acute outcome; no direct channel electrophysiology; male-only study	(61)
Incision increases DRG NaV1.7 with p-p65	SMIR; rat	Direct surgical model	NaV1.7 expression	Association	No channel electrophysiology	(57)
TNF-α/NF-κB modulates DRG NaV1.8 expression, neuronal excitability, and current properties after incision	Plantar incision; rat	Direct surgical model	NaV1.8 expression, VX-548-sensitive current properties, neuronal firing, and behavior	Pathway perturbation plus limited acute electrophysiology	Peak current increase after TNF-α was not significant; expression/current discordance; approximately 3–5 cells; no persistence or human correspondence	(62)
Incision activates spinal glia and IL-1β	Plantar incision; rat	Direct surgical model	Dorsal-horn glial and cytokine endpoints	Association	No nociceptor channel endpoint	(58)
Glial inhibition reduces postoperative hypersensitivity	Incision; rat	Direct surgical model	Mechanical hypersensitivity	Pharmacologic loss of function	Intervention lacks cell specificity	(59)
IL-1β relieves slow inactivation	DRG neurons	Non-surgical	TTX-resistant persistent/slow current	Direct electrophysiology	Human surgery not tested	(10)
TNF-α enhances TTX-resistant current through p38	DRG neurons; mouse	Non-surgical	Current density	Electrophysiology and TNFR1 loss of function	Incision not tested	(13)
p38 phosphorylates NaV1.8	DRG neurons	Non-surgical	NaV1.8 current density	Direct electrophysiology	Clinical correspondence unknown	(12)
TNF-α promotes NaV1.7 membrane insertion	Sensory neurons	Non-surgical	Compartment-specific trafficking	Direct cellular mechanism	Postoperative tissue not tested	(14)
TNF-α/NF-κB increases NaV1.7	Diabetic neuropathy; rat	Indirect	NaV1.7 expression and hypersensitivity	Pathway inhibition	Disease-model indirectness	(51)
IL-6/sIL-6R-gp130/JAK signaling increases CaV3.2	Spinal nerve ligation; rat	Indirect	CaV3.2 expression, function, neuronal excitability, and behavior	Trans-signaling inhibition and channel block	Nerve-injury model	(52)
CXCL13/CXCR5 increases NaV1.8 through p38	Inflammatory pain; mouse	Indirect	NaV1.8 current density	Electrophysiology and Cxcr5 loss of function	Incision not tested	(54)
DRG macrophages contribute to pain	Nerve injury; mouse	Indirect	Initiation and persistence	Cell depletion	No channel endpoint	(29)
Kir4.1 loss in satellite glia evokes pain-like behavior	Trigeminal ganglion; rat	Indirect	Pain-like behavior; neuronal hyperexcitability inferred	Targeted loss of function	No direct neuronal electrophysiology; different ganglion and no incision	(33)
Microglial P2X4-BDNF-KCC2 promotes disinhibition	Nerve injury; rat/mouse	Indirect	Central chloride-gradient shift	Loss of function	Central, non-surgical pathway	(35–37)
G9a represses potassium-channel genes	Nerve injury; rat/mouse	Indirect	Persistent transcriptional regulation	Loss of function	Does not prove postoperative reversibility	(63, 64)
Human NaV1.7 phenotype is pharmacologically reversible	Patient-derived iPSC nociceptors	Human, non-surgical	NaV1.7-dependent excitability	Functional pharmacology	Inherited disorder, not surgery	(100)
Peripheral transducers contribute to postoperative pain	Plantar incision; rat	Direct surgical model	ASIC3 inhibition/knockdown; TRPV1 and TRPA1 antagonism; modality-specific behavior	Functional and pharmacological evidence	Acute outcomes; distinct modalities and compartments; no proof of CPSP maintenance	(45–47)
Mast-cell mediators contribute to postoperative nociception	Plantar incision; mouse	Direct surgical model	Degranulation, histamine/serotonin release, mechanical allodynia	Pharmacological perturbation	Early pain; no direct NaV/CaV functional or chronic endpoint	(25)
Inflammatory suppression can impede pain resolution	Plantar incision with dexamethasone or diclofenac; mouse	Surgical model with induced failed resolution	Persistent mechanical hypersensitivity; prostaglandin-mediated recovery	Immune-pathway perturbation	Drug-induced phenotype; no channel-function endpoint or human causal evidence	(60)
SCN9A polymorphisms associate with postoperative pain	Hysterectomy cohort; human	Direct surgical population	CPSP association in 446 participants with chronic-pain data	Candidate-gene association	Common polymorphisms; analysis-dependent signals; not causal or population-attributable evidence	(49)

DRG, dorsal root ganglion; SMIR, skin/muscle incision and retraction; TNFR1, tumor necrosis factor receptor 1; TTX, tetrodotoxin.

## 2 Search approach and evidence framework

This narrative Review was based on an author-curated literature corpus and updated through targeted searches completed on July 30, 2026. PubMed/MEDLINE served as the primary biomedical database. Crossref and OpenAlex were used to verify citations and identify supplementary sources. The update specifically rechecked incision-specific immune–nociceptor studies, NaV1.8/NaV1.9 mechanisms, postoperative neuroimmune reviews, and literature published after the prior July 22 search.

During revision, we additionally examined the reviewer-identified literature on ASIC3, TRPV1, TRPA1, mast cells, inflammation-dependent pain resolution, and SCN9A associations, checking the cited reports through PubMed, Europe PMC, and publisher or preprint records. This targeted update was not an exhaustive rerun of the original search. Preprint evidence is explicitly identified and interpreted separately from peer-reviewed studies.

Searches combined terms for postoperative pain, CPSP, incision, nociceptors, dorsal root ganglia, neuroimmune signaling, cytokines, glial cells, and ion channels. Channel-specific terms included NaV1.7, NaV1.8, NaV1.9, CaV3.2, *SCN9A*, *SCN10A*, *SCN11A*, and *CACNA1H*. Separate searches addressed sex or gender and suzetrigine or VX-548. Each database was searched from inception to the final search date.

We included English-language primary studies that addressed human channel biology, human perioperative biomarkers, surgical-incision models, or functional neuroimmune regulation of the selected channels. Non-surgical neuropathic and inflammatory models were included only when they tested a mechanistic link relevant to the proposed framework. Systematic reviews, consensus reports, and major narrative reviews informed definitions, epidemiology, and clinical context.

We distinguished evidence type from directness to CPSP. Human genetic or clinical studies, incision-specific perturbation studies, functional studies in non-surgical models, and expression-only associations address different questions rather than forming a universal ranking. Rare human channelopathies can establish channel causality for a particular pain phenotype while remaining indirect evidence for postoperative chronification. Within each category, we assessed surgical context, acute versus persistent outcomes, cell specificity, functional measurement, pathway perturbation, temporal sampling, and agreement between target engagement and behavior. Expression-only changes, whole-tissue measurements, single-sex designs, and non-surgical extrapolation were treated as limitations.

The review was intentionally narrative because the studies varied substantially in species, tissue, intervention, timing, and outcome. We therefore did not calculate a pooled effect estimate or formal risk-of-bias score. Selection was purposive rather than exhaustive, and no systematic-review flow diagram was constructed. Each claim was calibrated to the most direct evidence supporting the relevant link.

## 3 Postoperative inflammation as a time- and compartment-dependent process

### 3.1 The acute-to-chronic transition is a trajectory, not a single threshold

Under the International Classification of Diseases, 11th Revision, CPSP is pain that develops or increases after surgery, persists beyond 3 months, and is not better explained by another cause (21). Its incidence varies by procedure and is often higher after operations that damage major sensory nerves (1, 22). Severe acute pain, preoperative pain, psychological vulnerability, and surgical factors consistently identify patients at greater risk (23, 24).

Hyperalgesic priming offers an experimental explanation for vulnerability before the 3-month threshold. An initial insult induces a persistent, use-dependent change in nociceptor function. In this state, a later stimulus evokes exaggerated and prolonged hypersensitivity (3, 4). Current models place this priming within a broader transition involving inflammation, neural plasticity, and failed resolution (5, 6).

Together, these observations support a putative perioperative and early subacute window during which some mechanisms may remain modifiable. Its duration is unknown and likely varies by procedure, baseline pain, sex-related biology, age, and the dominant mechanism. No validated bedside measure currently identifies the relevant immune and channel state in an individual patient.

For mechanistic analysis, the trajectory can be divided into overlapping phases. Tissue injury, nociceptor activation, and local inflammation dominate the immediate phase. Over subsequent days and weeks, persistent afferent input, altered ganglion and spinal signaling, and failed resolution may consolidate sensitization. Established CPSP is the clinical outcome of this trajectory rather than a single mechanism.

This distinction has direct implications for the design of clinical trials. Acute analgesia may reduce immediate pain without altering the later development of plasticity. Interventions targeting transcriptional regulation or immune resolution may require longer exposure and different outcomes. Trials should prespecify whether they target acute analgesia, priming prevention, consolidation interruption, or established pain.

### 3.2 Incision evokes insult-specific immune programs and resolution signals

Incision initiates immune programs at the wound, along injured or stressed afferents, within sensory ganglia, and in the spinal cord. These compartments overlap in time rather than forming a mandatory peripheral-to-central sequence. Neutrophils and macrophages can provide sensitizing mediators, while resident macrophages, satellite glia, endothelial cells, and spinal glia shape the extracellular environment encountered by nociceptor terminals and somata. The same compartments can also produce signals that limit sensitization or promote resolution, so cell identity, activation state, location, and postoperative time are as important as the concentration of any single cytokine.

Mast cells are another relevant wound-associated population. In a mouse plantar-incision study, mast-cell degranulation accompanied local histamine and serotonin release; mediator depletion or cromoglycate pretreatment reduced postoperative mechanical allodynia, and local receptor antagonism supported a contribution of these mediators (25). This pharmacological evidence concerns early postoperative nociception. It does not establish mast cells as a persistent immune source driving NaV or CaV remodeling in CPSP.

Single-cell analysis across zymosan inflammation, skin incision, and ultraviolet burn demonstrates that neuroimmune signaling is insult-specific rather than a generic inflammatory program. After skin incision, macrophage and neutrophil recruitment tracked the kinetics of pain, and ligand–receptor interactomes distinguished the incision response from other cutaneous injuries. The same study identified immune-cell-derived thrombospondin-1 as an inhibitor of nociceptor sensitization, providing direct evidence that postoperative immune activity contains both pain-promoting and pain-limiting components (26). This finding argues against a simple strategy of suppressing all inflammation and instead supports time- and cell-resolved targeting.

Neuroimmune signaling is not uniformly pathogenic, as endogenous IL-10 and CD8-positive T cells contribute to resolution in neuropathic pain (27). Regulatory T cells can also restrain microglia-associated hypersensitivity (28), reinforcing the need to distinguish harmful, time-dependent interactions from immune activity required for repair or resolution.

### 3.3 Peripheral, ganglion, and spinal compartments overlap

At the ganglion and spinal levels, evidence is less incision-specific but establishes plausible cellular sources and amplifiers of channel-regulating signals. The relevant question is not whether each compartment can influence pain, but which cell population is necessary at a defined postoperative time and whether its perturbation changes channel function in identified nociceptors.

Peripheral immune cells position inflammatory signals close to sensory neurons. DRG macrophages contribute to the initiation and persistence of neuropathic pain (29). Injury expands resident CX3CR1-positive macrophages that produce TNF-α and IL-1β (30), while Nox2-dependent redox communication links macrophages to sensory-neuron hypersensitivity (31).

A rat postsurgical model linked macrophage-endothelial Epac1-p120-catenin signaling to an altered perineuronal environment, but surgery-specific macrophage evidence remains limited (32). Kir4.1 disruption in satellite glia weakens potassium buffering and is sufficient to evoke pain-like behavior, although increased sensory-neuron excitability was inferred rather than measured directly (33). Connexin-mediated coupling can alter ganglion homeostasis (34), but direct satellite-glia-to-NaV gating has not been demonstrated after incision.

Central mechanisms may operate in parallel: after nerve injury, spinal microglial P2X4 receptors promote BDNF release (35, 36), and BDNF shifts the neuronal chloride gradient to facilitate disinhibition (37). In rats, incision also increases spinal BDNF and postoperative hypersensitivity (38). However, another incision study found that minocycline neither improved early mechanical thresholds nor suppressed spinal BDNF; it transiently reduced thermal withdrawal latency (39).

The peripheral, ganglion, and spinal compartments should not be viewed as a rigid sequence. Their contributions may arise in parallel and depend on the extent of tissue and nerve injury. **Figure 2** illustrates this overlap: a peripheral treatment may reduce central input without acting on spinal glia, while established central changes may outlast the initiating signal.

**Figure 2:**
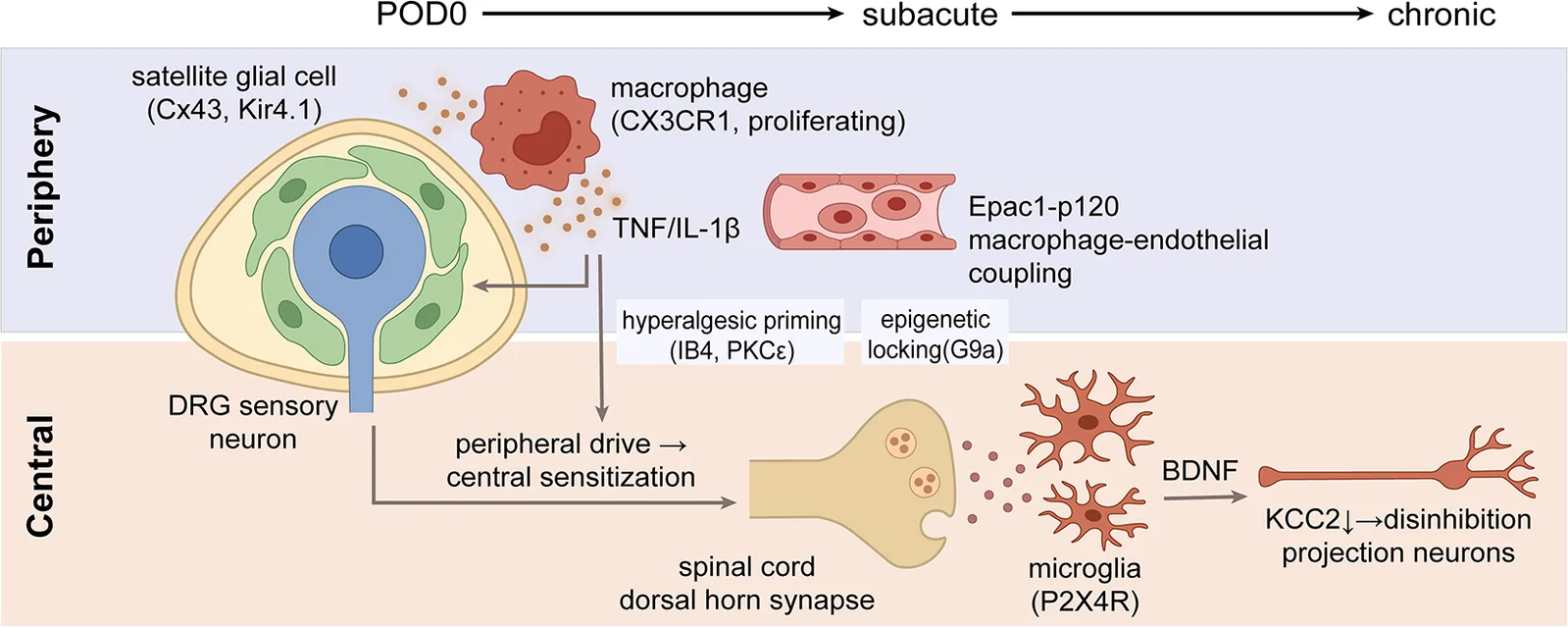
Parallel and overlapping engagement of peripheral terminals, DRG macrophages, satellite glia, and spinal microglia across the postoperative trajectory. The diagram is not intended to imply a fixed peripheral-to-central sequence. BDNF, brain-derived neurotrophic factor; Cx43, connexin 43; DRG, dorsal root ganglion; IB4, isolectin B4; KCC2, K+-Cl− cotransporter 2; P2X4R, P2X4 receptor; PKCϵ, protein kinase C epsilon; POD, postoperative day.

## 4 Nociceptor ion channels form a complementary excitability network

NaV1.7, NaV1.8, and NaV1.9 occupy complementary positions in nociceptor excitation. NaV1.7 amplifies slow depolarization near threshold, whereas NaV1.8 carries much of the action-potential upstroke and supports repetitive firing. NaV1.9 contributes a persistent subthreshold current, and CaV3.2 provides additional low-voltage gain (40).

Human genetics provides strong evidence for NaV1.7-dependent nociception in specific channelopathies: pathogenic gain-of-function SCN9A variants cause erythromelalgia or paroxysmal extreme pain disorder (8, 9) and can render sensory neurons hyperexcitable (41), whereas biallelic loss-of-function variants cause congenital insensitivity to pain (7, 42). These opposing phenotypes establish biological relevance, not the prevalence or attributable risk of NaV1.7-mediated CPSP.

NaV1.8 remains available at membrane potentials that inactivate many tetrodotoxin-sensitive channels. Genetic deletion impairs responses to noxious stimuli (43), whereas gain-of-function SCN10A variants are associated with painful neuropathy (44). NaV1.9 and CaV3.2 can lower the threshold for NaV1.8-dependent firing by altering subthreshold membrane behavior (40).

The network can be understood as a cascade of gain. NaV1.9 and CaV3.2 amplify small subthreshold inputs; NaV1.7 boosts slow depolarization near threshold; and NaV1.8 supports the action-potential upstroke and repetitive firing. Accordingly, a modest immune-induced change at one channel can recruit the others without producing a large change in total channel expression. This is why voltage dependence, inactivation, recovery, membrane trafficking, and current density should be reported together whenever possible.

This functional division explains both the promise and difficulty of channel-directed analgesia. Drug effects depend on state-dependent block, subtype selectivity, tissue exposure, and access to the relevant channel pool. Channel function can also vary with trafficking and post-translational signaling. Species differences preclude direct inference from rodent current density to human clinical effects.

Peripheral stimulus transduction should be distinguished from voltage-dependent amplification and firing. ASIC3, TRPV1, and TRPA1 provide relevant inputs to this excitability network. In rats, ASIC3 inhibition or knockdown reduced several postoperative pain behaviors, with increased ASIC3 expression after incision (45). TRPV1 antagonism reduced incision-induced heat hyperalgesia but not guarding or mechanical hypersensitivity in the cited study (46). TRPA1 antagonism reduced guarding and mechanical hypersensitivity, with different effects after peripheral and intrathecal administration (47). These results support modality- and compartment-dependent contributions rather than a single obligatory channel sequence. They concern acute postoperative pain and do not by themselves establish chronification. Our emphasis on NaV1.7–1.9 and CaV3.2 reflects their roles in voltage-dependent excitability and the available immune-to-channel functional studies; it does not imply that they are the only, or dominant, transducers after incision.

Subcellular location further constrains interpretation because somatic currents in dissociated DRG neurons may not represent channel density at peripheral, axonal, or central terminals. Cytokine-dependent trafficking can also differ among compartments (14), so incision studies should integrate kinetics, localization, and neuronal identity rather than rely on whole-ganglion messenger RNA.

The genetic evidence also requires population and phenotype calibration. In one primary erythromelalgia cohort, likely causal SCN9A variants were identified in 4 of 34 unrelated patients (48). A hysterectomy candidate-gene study evaluated three common SCN9A polymorphisms: among 446 participants with chronic-pain data, rs16851799 was associated with CPSP in univariate analysis and rs4387806 in multivariable analysis (49). These associations require replication and should not be conflated with rare, functionally established channelopathy mutations. A UK Biobank preprint reported no significant protective association with chronic pain for carriers of predicted loss-of-function variants in SCN9A, SCN10A, or SCN11A (50). These population findings temper extrapolation from rare channelopathies, although general chronic-pain phenotypes and predominantly heterozygous predicted variants do not directly test CPSP prevention or pharmacological channel inhibition. Collectively, these findings do not establish how much population-level CPSP susceptibility is attributable to NaV variation; failure to detect an association also does not exclude acquired immune regulation of the same channels.

## 5 Immune mediators reshape channel gating, trafficking, and expression

Inflammatory mediators regulate nociceptor channels across at least two timescales. Rapid post-translational signaling alters gating or membrane availability within minutes. Slower transcriptional pathways change channel abundance over hours or days. **Figure 3** summarizes these convergent mechanisms, most of which were established outside incision models.

**Figure 3:**
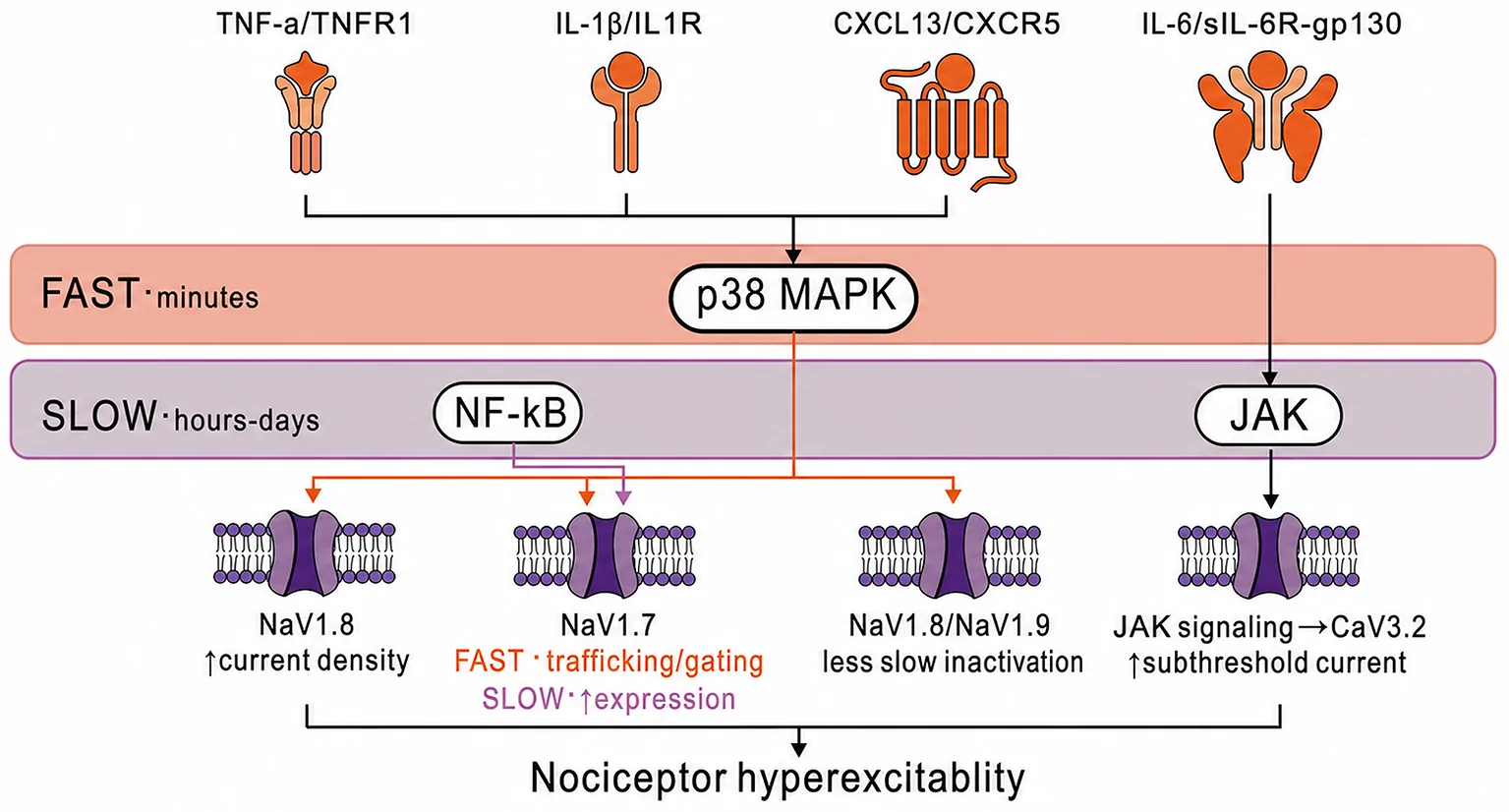
Cytokine and chemokine signaling through p38 MAPK, NF-κB, and gp130/JAK can alter NaV1.7, NaV1.8, NaV1.9, and CaV3.2 through post-translational and transcriptional mechanisms. Most depicted links are supported by non-surgical rodent or cellular studies. JAK, Janus kinase; MAPK, mitogen-activated protein kinase; NF-κB, nuclear factor kappa B.

IL-1β rapidly increases tetrodotoxin-resistant sodium current through p38-dependent relief of slow inactivation and enhancement of persistent current (10). TNF-α similarly increases tetrodotoxin-resistant sodium current through p38 MAPK signaling (13). Direct phosphorylation of NaV1.8 by p38 also increases current density (12).

TNF-α regulates NaV1.7 through distinct channel-proximal processes: acute exposure promotes compartment-specific membrane insertion in sensory neurons (14) and can shift sodium-current activation and inactivation (11). These findings support rapid control of channel availability and gating, but neither study used human postoperative nociceptors.

Sustained signaling can also engage transcription; in diabetic neuropathy, TNF-α acting through TNFR1 and NF-κB increased NaV1.7 expression. Pathway inhibition reduced both upregulation and hypersensitivity (51); rapid phosphorylation and delayed transcription remain plausible complementary mechanisms, but few studies have measured both longitudinally.

Fast and slow regulation should also be separated experimentally. Phosphorylation or trafficking can change excitability within minutes, whereas NF-κB- or JAK-dependent transcription typically evolves over hours to days. A measurement made at one postoperative time cannot distinguish these mechanisms, and an apparent increase in messenger RNA cannot be assumed to predict membrane current. Longitudinal studies should therefore pair molecular abundance with patch-clamp or other functional readouts at matched time points.

Beyond TNF-α, IL-6/sIL-6R trans-signaling increases CaV3.2 expression and function in DRG neurons through gp130/JAK signaling (52). CaV3.2 is also regulated during inflammatory hyperalgesia (53), while CXCL13 acts through CXCR5 and p38 MAPK to increase NaV1.8 current in inflammatory pain (54). Chemokine-mediated neuron-glia communication may therefore recruit several distinct channel effectors (55).

Pathway redundancy complicates intervention because TNF-α, IL-1β, IL-6, and chemokines do not form a single linear cascade. Their intracellular hubs also have many substrates beyond ion channels. Blocking one cytokine may leave convergent signaling intact, whereas broad kinase inhibition may affect immunity, hemostasis, and healing.

Cell identity remains uncertain in whole-DRG measurements that combine nociceptors, other sensory neurons, macrophages, satellite glia, endothelial cells, and connective tissue. A tissue-level increase may therefore reflect cellular composition rather than nociceptor regulation. Single-cell, spatial, and functional approaches are needed to link ligand exposure with channel behavior in the same cells.

## 6 What incision-specific studies establish—and what they do not

Incision models provide the most relevant test of the framework. The plantar-incision model reproduces guarding and mechanical hypersensitivity (56), whereas skin/muscle incision and retraction produces more persistent hypersensitivity and increased DRG NaV1.7 expression with phosphorylated p65 (57).

Incision activates spinal glia and increases dorsal-horn IL-1β (58), while pharmacologic glial inhibition reduces postoperative mechanical hypersensitivity in rats (59). These findings place neuroimmune activity after surgery, but they do not identify a common cellular mechanism across initiation, consolidation, and maintenance.

Plantar incision is best suited to model early postoperative hypersensitivity, whereas retraction models add sustained tissue stress and may reproduce selected persistent features. Neither captures the heterogeneity of human surgery, including pre-existing pain, anesthetic exposure, psychosocial risk, or multimorbidity. Replication across complementary models is therefore essential for reliable interpretation.

An additional model directly interrogates failed resolution. After plantar incision, saline-treated mice recovered within approximately 2 weeks, whereas dexamethasone treatment prolonged mechanical hypersensitivity beyond 10 weeks; diclofenac also prolonged recovery. Prostaglandin receptor agonists prevented or reversed the persistent phenotype, with perturbation experiments implicating neutrophils and PKA–STAT3–S100A8/A9 signaling (60). This pharmacologically induced model demonstrates that suppressing an early inflammatory response can impede recovery. It does not show that routine anti-inflammatory treatment causes human CPSP, or that protection requires normalization of NaV or CaV function. It therefore provides a complementary test of immune resolution rather than confirmation of the entire proposed channel-mediated sequence.

The most direct incision-specific pathway evidence predates the recent rat study. In male mice, plantar incision increased NaV1.8 and NaV1.9 transcripts in dorsal root ganglia, whereas antisense oligodeoxynucleotides targeting either channel reduced acute mechanical hypernociception. Inhibition of TNF-α, p38 MAPK, or NF-κB reduced both hypersensitivity and channel overexpression, while ERK and JNK antagonism did not reproduce the same pattern (61). This study links incision, an immune mediator, intracellular signaling, channel transcripts, and behavior in one model. Its limitations are equally important: interventions were largely preventive, animals were male, outcomes were acute, cell specificity was limited, and channel function was inferred from knockdown and expression rather than directly measured by electrophysiology.

A 2026 rat plantar-incision study adds neuronal excitability and VX-548-sensitive sodium-current measurements to the TNF-α/NF-κB/NaV1.8 model (62). TNF-α and NF-κB manipulations altered pain behavior, NaV1.8 expression, action-potential firing, and current properties. However, exogenous TNF-α did not significantly increase peak NaV1.8 current amplitude, expression and current changes were not uniformly concordant, and electrophysiology used approximately three to five cells per comparison and a single current-injection intensity. These data support pathway plausibility and functional modulation, but they do not establish a reproducible increase in NaV1.8 current or a persistent mechanism of CPSP.

Taken together, incision studies support several components of the proposed framework: immune-cell recruitment, cytokine and glial activation, NaV1.7/NaV1.8/NaV1.9 expression changes, channel-dependent acute hypersensitivity, and limited acute electrophysiology. The channel-focused studies do not yet demonstrate that a single immune-cell source drives a channel-gating change that persists after tissue healing, nor do they show that reversing that functional change prevents a chronic phenotype. The distinction matters because acute incision models can validate postoperative nociception while remaining poorly suited to the maintenance phase of CPSP.

Longer-lasting molecular regulation may stabilize a state initiated by inflammation and priming. After nerve injury, G9a-dependent repression of potassium-channel genes contributes to the acute-to-chronic transition (63, 64). MicroRNAs and DNA methylation also regulate transcriptional programs related to pain (65, 66). Targeted epigenetic repression of NaV1.7 produces prolonged analgesia in mice (67), but does not establish postoperative reversibility.

A decisive incision experiment would track channel function over time, interrupt the proposed immune pathway, and test persistent pain. Applying interventions at several postoperative times would distinguish initiation from maintenance. No human study has completed this sequence, and surgical animal evidence remains fragmentary.

## 7 Sex-, age-, and hormone-related modifiers

The framework should not be assumed to be sex-neutral because several rodent neuropathic and inflammatory models show similar hypersensitivity through different immune mechanisms in males and females (18, 68). **Figure 4** summarizes this preclinical architecture as a guide for stratified experiments, not a binary map of CPSP in women and men.

**Figure 4:**
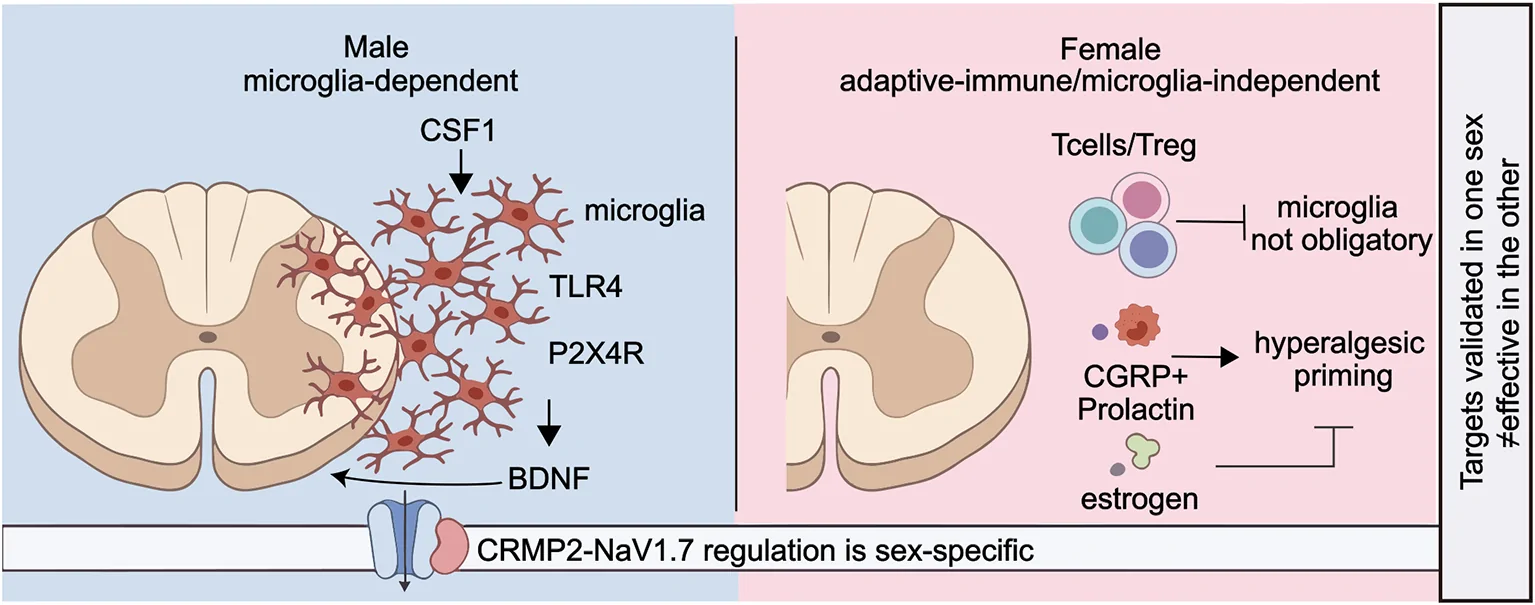
Model-dependent preclinical sex-related mechanisms involving microglia, adaptive immunity, neuroendocrine signaling, and CRMP2-dependent NaV1.7 regulation. The diagram summarizes selected rodent and cellular studies but does not establish corresponding binary pathways in human CPSP. BDNF, brain-derived neurotrophic factor; CGRP, calcitonin gene-related peptide; CRMP2, collapsin response mediator protein 2; CSF1, colony-stimulating factor 1; P2X4R, P2X4 receptor; TLR4, Toll-like receptor 4; Treg, regulatory T cell.

Male rodents preferentially recruit spinal microglial pathways in specific models. Under these conditions, TLR4 and microglial P2X4 signaling are required for hypersensitivity in males but not females (69, 70). Injured-neuron-derived CSF1 can drive upstream microglial expansion (71), although the direction and magnitude of these effects depend on model, outcome, and timing.

T-cell deficiency changes pain sensitivity and opioid analgesia (72), and adaptive immune cells rather than microglia maintain mechanical hypersensitivity in female mice in specific neuropathic models (18). Regulatory T cells restrain microglia-associated hypersensitivity (28), while T cells and IL-10 contribute to resolution (27). Estrogen also modifies glial activation (73); together, these findings describe interactions among immune availability, hormonal context, and pain phase rather than a universal female pathway.

Sex differences affect nociceptor priming: inhibiting spinal P2X receptors or p38 disrupts priming in male but not female mice (74). Nociceptor αCaMKII contributes differently by sex in rats (75), while prolactin-receptor and CGRP signaling provide female-biased routes to priming (76, 77). In one transgenic model, CRMP2 SUMOylation produces sex-dependent effects on NaV1.7 regulation (17).

Surgery-specific evidence is beginning to refine this picture: age and sex altered behavioral and neuroimmune phenotypes after incision in rats (78). A 2025 study linked stress and prolactin signaling to nociceptor sensitization and prolonged postoperative hypersensitivity in female mice. It also showed prolactin-dependent sensitization in female human nociceptors, but did not test chronic postsurgical outcomes in patients (79).

Single-cell studies show time- and sex-dependent microglial transcriptional responses after nerve injury (80, 81), but their human correspondence remains unresolved. Female sex has been associated with CPSP in some cohorts and reviews (23, 24, 82, 83), although estimates vary by operation and potential confounding from preoperative pain, age, psychosocial factors, and hormonal state.

Biological sex is not equivalent to stable hormone exposure because menstrual phase, pregnancy, menopause, exogenous hormones, and aging can modify immune and neuronal responses. Sex-related biology must also be distinguished from gender-related exposures, treatments, and reporting. Thus, a clinical association with sex cannot validate a microglial, T-cell, or prolactin mechanism.

The strongest near-term implication is methodological: preclinical experiments should include both sexes, appropriate ages, and prespecified interaction tests. Clinical studies should prospectively collect relevant hormonal and gender-related variables. Mechanistic claims should require an intermediate biomarker or a prospectively specified treatment-by-sex interaction.

This literature should be interpreted as evidence of context dependence, not as two fixed male and female pathways. Immune-cell requirements vary by species, injury model, tissue, age, and hormonal milieu, and several influential observations rest on single studies. Future experiments should report estrous or hormonal status when relevant, include aged animals, and test statistical interactions rather than infer dimorphism from significance in one sex and non-significance in the other.

## 8 Human translation: tractable channel targets, unresolved disease modification

**Table 2** summarizes the clinical evidence for channel-directed and selected perioperative interventions. Suzetrigine provides the clearest clinical validation of the peripheral channel node. In phase 2 trials after abdominoplasty and bunionectomy, only the highest-dose VX-548 regimen reduced 48-hour acute pain versus placebo (20). Two phase 3 trials supported efficacy and tolerability after bunionectomy and abdominoplasty (19). A single-arm phase 3 study extended experience to acute surgical and non-surgical pain (84).

**Table 2:** Clinical evidence for channel-directed and selected perioperative interventions.

Intervention	Target	Clinical context	Acute pain evidence	CPSP prevention evidence	Interpretation	Ref.
Suzetrigine	NaV1.8	Phase 2 trials after abdominoplasty and bunionectomy	Only the highest-dose regimen reduced acute pain over 48 hours versus placebo; lower doses did not	Not tested	Validates acute target tractability; dose dependence limits generalization	(20)
Suzetrigine	NaV1.8	Two phase 3 surgical trials	Supported acute efficacy and tolerability	Not tested	Acute regimens cannot be assumed to prevent CPSP	(19)
Suzetrigine	NaV1.8	FDA-approved treatment for moderate-to-severe acute pain in adults	Approved for moderate-to-severe acute pain	No prevention indication	Approval does not establish CPSP modification	(85)
PF-05089771	NaV1.7	Phase 2 trial in painful diabetic neuropathy	Primary criterion not met	Not applicable	Genetic validation did not ensure efficacy	(87)
Selective NaV1.7 blocker	NaV1.7	Phase 2a trial in trigeminal neuralgia	Limited numerical benefit	Not applicable	Exposure and state dependence remain concerns	(88)
Vixotrigine	Use-dependent sodium-channel block	Phase 2 trial in small-fiber neuropathy	Mixed dose response	Not applicable	Target and dose relationships require clarification	(89)
Pregabalin/gabapentinoids	α2δ subunit	Perioperative meta-analyses	Acute effects vary	Evidence does not support routine CPSP prevention	Distinguishes acute analgesia from CPSP prevention	(91, 92)
Ketamine	NMDA receptor	Perioperative systematic reviews	Acute benefit is better established	Evidence for persistent-pain prevention remains uncertain	Timing and heterogeneity limit inference	(94, 95)

CPSP, chronic postsurgical pain; FDA, US Food and Drug Administration; NMDA, N-methyl-D-aspartate.

The US Food and Drug Administration approved suzetrigine in January 2025 for moderate-to-severe acute pain in adults (85). A subsequent meta-analysis found modest reductions in acute postoperative pain at 24 and 48 hours versus placebo (86). These findings establish tractability for acute analgesia, not prevention of CPSP or modification of neuroimmune reprogramming.

The suzetrigine program illustrates the distinction between target validation and prevention. Acute trials use short treatment intervals; CPSP trials require follow-up beyond 3 months and sufficient power for a less frequent outcome. Acute-pain regimens cannot be assumed to suppress priming or transcriptional consolidation.

NaV1.7 development provides a counterpoint: PF-05089771 did not meet its primary efficacy criterion in painful diabetic neuropathy (87). A selective blocker provided limited benefit in trigeminal neuralgia (88), while vixotrigine showed an equivocal dose response in small-fiber neuropathy (89). Exposure, state dependence, and access to relevant channel pools may partly explain the gap between genetic validation and clinical efficacy (90).

Perioperative prevention trials are similarly cautionary: early analyses suggested that gabapentinoids might reduce CPSP (91), but later evidence did not support routine pregabalin prevention (92). Broader pharmacotherapy reviews remain inconclusive (93), and ketamine findings differ between persistent- and acute-pain syntheses (94, 95). Regional and procedure-specific strategies improve acute care but do not establish channel-specific prevention (96, 97).

Emerging human biomarker evidence remains associative: perioperative cytokine patterns differed between acute pain and CPSP after pediatric major musculoskeletal surgery (98). An exploratory knee-arthroplasty analysis found that baseline cytokines modified associations between intraoperative oxidative stress and 6-month pain (99). Neither study measured ion-channel function, and both findings require replication.

Human evidence therefore anchors the framework most strongly at its channel end. Patient-derived induced pluripotent stem cell nociceptors reproduce an NaV1.7 pain phenotype and respond to selective blockade (100). Spatial transcriptomics maps channel and receptor expression in human DRG and reveals species differences (101). The intervening cytokine-to-channel sequence remains largely untested in human perioperative tissue.

Human evidence therefore supports distinct components of the framework rather than a continuous causal sequence. Channelopathies and patient-derived nociceptors establish that particular NaV abnormalities can cause pain, whereas acute NaV1.8 trials establish pharmacological analgesia and perioperative cytokine studies identify associations. None establishes the population contribution of NaV variants to CPSP. The mechanistic gap remains: human surgical tissue has not shown that a defined immune-cell population activates p38 MAPK or NF-κB in identified nociceptors, changes channel gating or trafficking, and predicts pain beyond three months.

Closing this gap will require paired human systems. Surgical tissue or blood can define an immune signature, while induced pluripotent stem cell nociceptors, freshly procured human dorsal root ganglia, or ex vivo sensory-neuron assays can test whether the same signature changes channel function. Spatial and single-cell methods should identify ligand-producing immune cells and receptor-bearing nociceptor subtypes, but causal inference will still require perturbation, functional recording, and longitudinal clinical outcomes.

## 9 Discussion

### 9.1 What the evidence establishes

The current literature supports conclusions at different levels. Pathogenic sodium-channel variants can cause specific human pain phenotypes, and selective NaV1.8 inhibition provides acute analgesia. Experimental systems show that immune mediators can modify channel gating, trafficking, and expression. Incision activates relevant immune compartments and can alter channel expression. These observations support mechanistic plausibility, but do not establish a major contribution of inherited NaV variation to CPSP or demonstrate that channel blockade prevents chronification.

The most informative direct surgical study is the mouse TNF-α/p38 MAPK/NF-κB/NaV1.8–NaV1.9 perturbation experiment (61), while the 2026 rat study contributes preliminary acute electrophysiology (62). Neither establishes persistence, human correspondence, or a complete immune-cell-to-channel causal chain. Most detailed gating and trafficking mechanisms still derive from inflammatory, diabetic, neuropathic, or cellular preparations.

Postoperative inflammation and channel dysregulation could instead be parallel consequences of tissue injury. Channel changes might also drive only acute nociception, with later pain maintained by other mechanisms. Co-occurring cytokine and transcript changes alone cannot exclude either explanation. Resolving these alternatives requires direct, time-specific perturbation and rescue experiments.

The failed-resolution model further suggests that an intact early immune response can protect against persistent postoperative hypersensitivity (60). Non-neuronal cells may help determine whether pain resolves, while neuronal channels participate in nociceptive output; these roles need not be mutually exclusive. However, assigning primacy to either compartment requires direct comparison of cell-specific interventions during initiation and maintenance. It also remains necessary to test whether an immune intervention changes identified nociceptor currents or acts through other routes. Thus, prostaglandin-mediated protection is evidence for a resolving immune mechanism in that model, not proof that neuronal channel remodeling is dispensable in human CPSP.

The framework should therefore be interpreted as a causal map with graded edges. Human genetics and acute pharmacology support the channel node, while incision studies anchor the framework in a postoperative context. The weakest edge remains the longitudinal neuroimmune-to-channel mechanism that would predict CPSP in humans.

Three controversies organize the field. First, peripheral, ganglion, and spinal mechanisms may be sequential in some models but parallel in others. Second, postoperative immunity is simultaneously sensitizing and pro-resolving, so the direction of effect depends on cell state and time (26–28). Third, an intervention that reduces acute channel-dependent pain may or may not modify the biological transition to CPSP. These alternatives yield different predictions and should be treated as competing models rather than combined into an assumed linear cascade.

### 9.2 The smallest testable causal sequence

A useful next experiment need not reproduce every arrow in the framework. The smallest testable sequence has three steps: incision changes a defined immune signal; that signal alters channel function in identified nociceptors; the functional change contributes to persistent pain. Each step should be measured in the same model and over the same time course.

Establishing causality requires blocking the immune signal to prevent the channel change and reduce persistent pain. When technically feasible, restoring the downstream channel effect should rescue the phenotype. Intervening after pain develops should determine whether the mechanism controls maintenance or only initiation.

Human translation requires a measurable intermediate, such as blood or surgical-tissue signatures, quantitative sensory testing, or patient-derived nociceptor assays. A biomarker need not measure gating directly, but it should identify pathway activity and change in the predicted direction with treatment.

Sex-related biology should be prespecified as a modifier of this sequence rather than added as a *post hoc* explanation. The relevant question is whether sex or hormonal context alters target engagement or treatment response. Differences in pain prevalence alone cannot identify the responsible cellular pathway.

The framework makes falsifiable predictions. If an immune-to-channel mechanism contributes to chronification, then blocking the immune signal after incision should prevent or normalize a measured channel-function change, and channel-specific rescue should restore the relevant pain phenotype. If the mechanism is limited to initiation, delayed intervention after hypersensitivity is established should fail despite acute target engagement. If immune resolution is the dominant determinant, broad early suppression may prolong rather than shorten the pain trajectory. Testing these predictions across sex, age, and postoperative time would distinguish mechanism from association.

### 9.3 A target–time–population–biomarker–outcome framework

Mechanism-based prevention requires alignment across target, timing, population, biomarker, and outcome. The target must be active during treatment, and the population must have sufficient CPSP risk and evidence of target relevance. A biomarker should support target engagement, while the outcome must distinguish acute analgesia from an altered pain trajectory.

For a channel blocker, acute pain and opioid use are informative but insufficient. Follow-up beyond 3 months should assess pain intensity, interference, neuropathic features, analgesic use, and recovery trajectory. Repeated measurements can more clearly distinguish delayed resolution from persistent pain.

For an upstream immune or kinase intervention, safety is inseparable from mechanism. The same pathway may affect host defense, hemostasis, healing, or resolution. Local or sensory-neuron-selective delivery may improve specificity, but these strategies remain experimental.

Suzetrigine makes prevention studies timely, but its acute efficacy does not define an effective CPSP regimen. A prevention trial requires a biological rationale for treatment timing and duration. It should also specify whether the intended benefit is symptom control, prevention of priming, or interruption of consolidation.

### 9.4 Limitations

Indirectness across the proposed causal links is the principal limitation of this review. Human genetics and channel-blocker studies support channel biology (7–9, 19, 20, 41–44, 84, 87–90, 100), but most functional immune-to-channel links arise from non-surgical models, and incision-specific evidence remains limited (38, 39, 56–59, 62).

The synthesis is narrative rather than systematic, allowing linkage across heterogeneous models but increasing vulnerability to selection and publication bias. Searches were restricted to English-language reports, and no formal risk-of-bias instrument was applied. **Table 1** therefore displays directness and evidence class so readers can judge the proposed continuity.

Sex-related evidence is especially vulnerable to overtranslation because most mechanistic studies used young rodents and binary comparisons. Human hormonal state, age, gender-related exposures, procedure, and psychosocial factors are more complex (17, 22, 73, 80–83, 102). The review emphasizes four voltage-gated channels while placing them alongside selected peripheral transducers; it cannot comprehensively cover all channels or the synaptic, psychological, and social drivers of CPSP.

Finally, durable epigenetic regulation does not establish the reversibility of established postoperative pain (63–67). The absence of an identified human mechanism likewise does not prove that none exists. The framework should be tested against its explicit predictions and revised as direct surgical and human evidence accumulates.

## 10 Conclusion

Neuroimmune regulation of nociceptor ion channels is a plausible contributor to postsurgical pain chronification, but it is not an established human surgical pathway. Rare channelopathies and acute pharmacology support channel relevance without establishing population-level genetic susceptibility or disease modification. Incision studies also identify peripheral transducers and immune mechanisms that can promote pain resolution. The contribution of channel remodeling must therefore be evaluated alongside these complementary and potentially competing explanations.

The central gap is the causal sequence linking incision, a defined immune signal, altered channel function, and persistent pain. Closing this gap requires incision-specific perturbation studies, human nociceptor systems, longitudinal biomarkers, and trials aligned across target, timing, population, biomarker, and outcome. Suzetrigine establishes tractability for acute analgesia, not prevention of CPSP. Its success should motivate rather than pre-empt a direct test of prevention.
